# Myricetin improves endurance capacity by inducing muscle fiber type conversion via miR-499

**DOI:** 10.1186/s12986-019-0353-8

**Published:** 2019-05-02

**Authors:** Luting Wu, Li Ran, Hedong Lang, Min Zhou, Li Yu, Long Yi, Jundong Zhu, Lei Liu, Mantian Mi

**Affiliations:** Research Center for Nutrition and Food safety, Chongqing Key Laboratory of Nutrition and Food Safety, Institute of Military Preventive Medicine, Third Military Medical University, Chongqing, China

**Keywords:** Myricetin, Endurance, Muscle fiber type, Myosin heavy chain, miR-499

## Abstract

**Background:**

Reprogramming of fast-to-slow myofiber switch can improve endurance capacity and alleviate fatigue. Accumulating evidence suggests that a muscle-specific microRNA, miR-499 plays a crucial role in myofiber type transition. In this study, we assessed the effects of natural flavonoid myricetin on exercise endurance and muscle fiber constitution, and further investigated the underlying mechanism of myricetin in vivo and in vitro.

**Methods:**

A total of 66 six-week-old male Sprague Dawley rats were divided into non-exercise or exercise groups with/without orally administered myricetin (50 or 150 mg/kg) for 2 or 4 weeks. Time-to-exhaustion, blood biochemical parameters, muscle fiber type proportion, the expression of muscle type decision related genes were measured. Mimic/ inhibitor of miR-499 were transfected into cultured L6 myotubes, the expressions of muscle type decision related genes and mitochondrial respiration capacity were investigated.

**Results:**

Myricetin treatment significantly improved the time-to-exhaustion in trained rats. The enhancement of endurance capacity was associated with an increase of the proportion of slow-twitch myofiber in both soleus and gastrocnemius muscles. Importantly, myricetin treatment amplified the expression of miR-499 and suppressed the expression of Sox6, the down-stream target gene of miR-499, both in vivo and in vitro. Furthermore, inhibition of miR-499 overturned the effects of myricetin on down-regulating Sox6.

**Conclusions:**

Myricetin promoted the reprogramming of fast-to-slow muscle fiber type switch and reinforced the exercise endurance capacity. The precise mechanisms responsible for the effects of myricetin are not resolved but likely involve regulating miR-499/Sox6 axis.

## Introduction

Skeletal muscle is composed of myofibers with different contractile and metabolic properties. The efficiency of muscle contraction depends on gene expression of two isoforms of myosin heavy chain (MHC), *α-MHC* and *β-MHC* (also known as *Myh6* and *Myh7*, respectively). Slow-twitch fibers, also known as type I myofiber, predominantly express *β-MHC*, display oxidative metabolism and high endurance [1]. Evidences have shown that the proportion of type I myofiber correlated with insulin sensitivity [2, 3]. Type I fibers have higher glucose-handling capacity compared with type II fibers, due to higher expressions of insulin receptor, GLUT4, hexokinase II and glycogen synthase [4]. Under certain physiological or pathological stimuli, such as exercise or obesity, the constitution of myofibers swifts from fast-to-slow or slow-to-fast, which are referred to as skeletal muscle plasticity [5].

MicroRNAs (miRNAs) represent a family of noncoding RNA of 21–22 nucleotides in length that regulate the expression of gene involved in developmental and cellular processes, such as skeletal muscle plasticity. Myosin-encoded miRNAs, also known as MyomiRs, have the ability to regulate myosin switching and myofiber identity [6]. Encoded by intron 19 of *Myh7b* gene, miR-499 is co-expressed with *β-MHC* and almost exclusively expressed in slow-twitch myofiber. miR-499 played a crucial role in skeletal muscle fast-to-slow reprogramming via suppressing the expression of transcriptional repressors of slow-twitch myofiber gene, such as Sox6 [7, 8]. Research found that the 3^′^ UTR of Sox6 mRNA contained four conserved target sites for miR-499, which meant that Sox6 was one of the down-stream target genes of miR-499 to modulate muscle fiber type specification and muscle performance [1].

Undoubtedly, exercise is beneficial to enhance physical performance and improve metabolic status. Evidences showed that endurance exercise promoted skeletal muscle fast-to-slow shift while disuse led to slow-to-fast shift [9, 10]. However, exercise may not be practical under certain circumstances, such as physical limitations. Therefore, it’s essential to explore exercise substitutes/mimetics that can achieve analogous health benefits of regular exercise [11, 12]. Recently, synthetic drugs, such as AMPK and PPARβ agonists (AICAR and GW501516, respectively) were found to enhance the proportion of slow-twitch myofiber and amplify mitochondrial function. Although, these drugs are indeed potent to improve endurance capacity, they cannot be applied to human trials due to the uncertainty of their safety [13].

Natural flavonoids possess a wide range of health benefits including promoting physical performance, anti-diabetic and anti-obesity effects [14, 15]. Flavonoid myricetin, derived from vegetables and fruits [16, 17], possesses numerous bioactivities, including anti-inflammatory, anti-diabetic and anti-carcinogen effects [18, 19]. Our previous study revealed that myricetin improved physical performance under hypoxic environment via maintaining mitochondrial biogenesis [20]. Recently myricetin was found to enhance mitochondrial activity to improve physical endurance [21]. However, the precise mechanism for endurance enhancement by myricetin has yet to be fully elucidated.

Here, we investigated the effects of myricetin on endurance capacity and myofiber type transition in SD rats, and explored the underlying mechanisms for these effects in rat L6 myotubes. We illustrated the role of miR-499/Sox6 pathway in myricetin-induced skeletal muscle reprograming and endurance enhancement.

## Materials and methods

### Chemicals and reagents

Myricetin (DY0103, HPLC ≥98%) was purchased from MUST Biotechnology Co., Ltd. (Chengdu, China) for animal study. For cell experiments, myricetin (70050) and DMSO (D2650) was obtained from Sigma-Aldrich (St. Louis, MO, USA). Dulbecco’s modified Eagle medium (DMEM) and horse serum (16050130) were bought from Gibco (Carlsbad, CA). Fetal bovine serum (FBS) was purchased from Hyclone Laboratories, Inc. (Logan, UT, USA). Cell Counting Kit-8 (CCK-8) was purchased from Dojindo (Kumamoto, Japan). mirVana™ miRNA Inhibitors (rno-miR-499-5p, MH11352), mirVanaTM miRNA mimics (rno-miR-499-5p, MC11352), Lipofectamine RNAiMAX Transfection Reagent (13778083) and antibody against PGC-1α (PA5–38021) were bought from Invitrogen (Massachusetts, USA). Antibodies against slow skeletal myosin heavy chain (ab11083), fast skeletal myosin heavy chain (ab91506), Sox6 (ab30455), tnni1 (ab231720) and myoglobin (ab77232) were purchased from Abcam (Cambridge, UK). Antibody against Cytochrome C (Cyt C, sc-13,561) and β-actin (sc-47778) were purchased from Santa Cruz Biotechnology (CA, USA).

### Animals and experimental design

A total of 66 six-week-old male Sprague Dawley (SD) rats were purchased from and housed three per cage under controlled conditions of temperature (22 ± 2 °C), humidity (60 ± 5%) and 12 h light/dark cycle in the specific pathogen-free grade room of the Experimental Animal Center of the Army Medical University (Chongqing, China). Food and water were freely available. All protocols involving animals and their care were performed according to institutional guidelines with the approval of the Institutional Animal Care and Use Committee of the Army Medical University.

After one week of acclimatization, 66 male rats were randomized into eleven groups (*n* = 6 per group): non-exercise group (NE), 50 mg/kg myricetin for 2 weeks (NE-2-MYR50), 150 mg/kg myricetin for 2 weeks (NE-2-MYR150), 50 mg/kg myricetin for 4 weeks (NE-4-MYR50), 150 mg/kg myricetin for 4 weeks (NE-4-MYR150), exercise training for 2 weeks (Ex-2), exercise training for 4 weeks (Ex-4), exercise training for 2 weeks with 50 mg/kg myricetin (Ex-2-MYR50), exercise training for 2 weeks with 150 mg/kg myricetin (Ex-2-MYR150), exercise training for 4 weeks with 50 mg/kg myricetin (Ex-4-MYR50), exercise training for 4 weeks with 150 mg/kg myricetin (Ex-4-MYR150). After dissolved in distilled water (2 mL), myricetin was given by orally gavage once a day, while the other groups were treated with the equal volume of distilled water.

### Exercise training and run-to-fatigue experiment

One hour after vehicle or myricetin administration, animals in the Ex-2, Ex-4, Ex-2-MYR50, Ex-2-MYR150, Ex-4-MYR50, Ex-4-MYR150 groups performed a regular running on a motorized treadmill at speed of 20 m/min. They were trained 20 min on the first day, and then increased by 10 min each day, it will not increase when it reached to 60 min, 5 days/week.

At the end of intervention period, all groups performed a strenuous running on the treadmill at a speed of 25 m/min. Exhaustion was determined as the inability to run despite continuous electronic shocking for 20 s. The time from beginning to exhaustion is defined as the time-to-exhaustion (TTE) that is used to assess endurance capacity.

Immediately after the run-to-fatigue exercise, venous blood was collected from the tail of the rats to monitor blood glucose and lactate by Onetouch Ultra (Johnson, USA) and Lactate Scout (EKF, Germany), respectively. After euthanized, soleus (SOL), gastrocnemius (GAS) muscles and liver were quickly removed, washed with saline, and stored at − 80 °C for further analyses.

### Immunofluorescence staining

Fresh soleus and gastrocnemius muscles were fixed in 4% paraformaldehyde at 4 °C 24 h, dehydrated with 30% sucrose solution, embedded in paraffin, and then sectioned with 5-μM thickness for immunofluorescence.

Sections were successively immerged into xylene for 15 min, replacing the xylene for another 15 min, anhydrous ethanol for 5 min, replacing the anhydrous ethanol for another 5 min, 85% ethanol for 5 min, and 75% ethanol for 5 min. Then sections were placed in a repairing kit filled with EDTA antigen retrieval buffer (pH 8.0). After blocking in BSA for 30 min, sections were incubated at 4 °C overnight in primary antibodies against slow skeletal myosin heavy chain (1:100) and fast myosin skeletal heavy chain (1:100). The next day, sections were washed with PBS three times followed by incubation with fluorescent-labeled secondary antibodies for 1 h. Then sections were incubated in DAPI Staining Solution for 10 min, washed with PBS for three times, mounted with Antifade Mounting Medium and photographed under MultiPhoto Laser Scanning Microscopy (Zeiss LSM780NLO).

### RNA extraction, reverse transcription and real-time PCR

Total RNA was extracted from soleus, gastrocnemius muscles, or L6 myotubes using the Trizol reagent (Invitrogen) according to the manufacturer’s instructions. For real-time PCR, RNA was retrotranscribed using PrimeScript RT Master Mix (Takara) according to the manufacturer’s directions. Gene-specifc primers were designed by Sangon Biotech (Shanghai) Co., Ltd. Real-time PCR was conducted with TB Green Premix Ex Taq II (Takara) according to manufacturer’s instructions. The mRNA expressions were normalized to those of GAPDH used as endogenous control. Gene specifc primer sequences are listed in Table 1.

**Table 1:** Primers used for Real-time PCR

Gene		Primer sequence	Ta(°C)
Myh1	Forward	GCGACAGACACCTCCTTCAAGAAC	60
	Reverse	CCAGCCAGCCAGCGATGTTG	
Myh4	Forward	CCATCACTGACGCCGCCATG	60
	Reverse	GTTCTTCTTCATCCGCTCCAGGTG	
Myh7	Forward	GAGCAGGAGCTGATCGAGAC	60
	Reverse	ATGGCCTTCTTGGCCTTCTC	
Myh7b	Forward	CAACCTGGCTAAGTACCGCA	60
	Reverse	AATAAGGACAGGACAGCGGC	
Myoglobin	Forward	CACCATGGGGCTCAGTGATG	60
	Reverse	CTCAGCCCTGGAAGCCTAGC	
PGC-1α	Forward	GCAGCGGTCTTAGCACTCA	60
	Reverse	GACTGCGGTTGTGTATGGGA	
Sox6	Forward	TCCATGGCTTTGTCACTTTCA	60
	Reverse	ATACACGAGGATCACCCCAG	
GAPDH	Forward	ACGGGAAGCTCACTGGCATGG	60
	Reverse	CGCCTGCTTCACCACCTTCTT	

### TaqMan miRNA

Total RNA was extracted from soleus, gastrocnemius muscles or L6 myotubes using the Trizol reagent (Invitrogen) according to the manufacturer’s instructions. TaqMan methods were used to assess miR-499 expression. 1 μg RNA was reverse transcribed to cDNA with the TaqMan® MicroRNA RT Kit (Applied Biosystems™). Thermal-cycling conditions of reverse transcription: 16 °C 5 min, 42 °C 50 min, 85 °C 5 min. Product from RT reaction was used to Quantitative Real-time PCR with the TaqMan MicroRNA Assays (Applied Biosystems™) specific for miR-499 (Assay ID: 472838_mat) and miR-U6. Thermal Cycling Conditions of qPCR: 94.5 °C 10 min for 1 cycles, 97 °C 30s and 59.7 °C 1 min for 40 cycles. The relative miR-499 level was normalized to miR-U6.

### Western blot

The protein was extracted from soleus, gastrocnemius muscles, and L6 myotubes. Briefly, protein samples were electrophoretically separated by 10% SDS-PAGE, then transferred to PVDF membranes, blocked for 1 h at room temperature using 5% blocking solution, incubated with primary antibodies: anti-*Myh7* (1:1000), anti-Sox6 (1:1000), anti-tnni1 (1:1000), anti-myoglobin (1:1000), anti-cytochrome c (1:1500), and anti-PGC-1α (1:1500) overnight at 4 °C. After incubation with relative secondary antibodies for 1 h at room temperature, signal development was detected using Immobilon Western Chemiluminescent HRP Substrate (Millipore). Images were aquired using fusion fx5 molecular imager (Vilber Lourmat).

### Cell culture and treatments

Rat skeletal muscle L6 myoblasts were obtained from the Cell Bank of Chinese Academy of Sciences (Shanghai, China) and were grown in DMEM containing 10% FBS and 2% penicillin-streptomycin in 5% CO_2_ at 37 °C. Cells were induced to L6 myotubes by the differentiation medium containing DMEM and 2% horse serum. When the myoblasts were fully differentiated to myotubes, they were treated with myricetin (2 μM, 10 μM, and 50 μM) or vehicle (DMSO) for 24 h.

### Cell proliferation assay

CCK-8 kits were used to measure cell proliferation following the manufacturer’s protocol. Briefly, L6 myoblasts were seeded in 96-well plates (5000 cells/well) and differentiated to myotubes. After fully differentiated, myotubes were incubating with myricetin (1-64 μM) or 0.1% DMSO as control for 24 h. Subsequently, CCK-8 solution (10 μL/well) was added to each well of the plates, and the plates were incubated for 1 h in the incubator. Cell viability was detected by the absorbance at 450 nm using an Infinite™ M200 Microplate Reader (Tecan, Mannedorf, Switzerland).

### Manipulation of miR-499 in L6 myotubes

To modulate the expression of miR-499, inhibitor and mimic were transfected into L6 myotubes at the final concentration of 10 nM using Lipofectamine™ RNAiMAX Transfection Reagent according to the manufacturer’s instructions. Twenty-four hours after transfection, myotubes were treated with 10 μM myricetin or vehicle (DMSO) for 24 h.

### Seahorse XFp cell mito stress test

Cell Mito Stress Test was measured using XFp Analyzer (Seahorse Bioscience, Agilent Technologies, USA). L6 myoblasts were seeded and differentiated in XFp Cell Culture Microplates. After differentiation and treatment, L6 myotubes were cultured in a 37 °C non-CO_2_ incubator for 1 h with assay medium, which contained 10 mM glucose, 1 mM pyruvate, and 2 mM glutamine, and were adjusted to pH 7.4 at 37 °C. Oligomycin, FCCP, and rotenone/antimycin were loaded in sensor cartridge for Cell Mito Stress Test. All steps were in accordance with the manufacturer’s instructions.

### Statistics

Data are represented as mean ± SD. Statistical analyses were conducted by one-way ANOVA, followed by Tukey’s test using GraphPad prism 6.0 (GraphPad Software, Inc., La Jolla, CA, USA). *P-*values less than 0.05 were considered to be statistically significant. All experiments were repeated a minimum of three times.

## Results

### Myricetin improved the aerobic capacity in rats

**Fig. 1:**
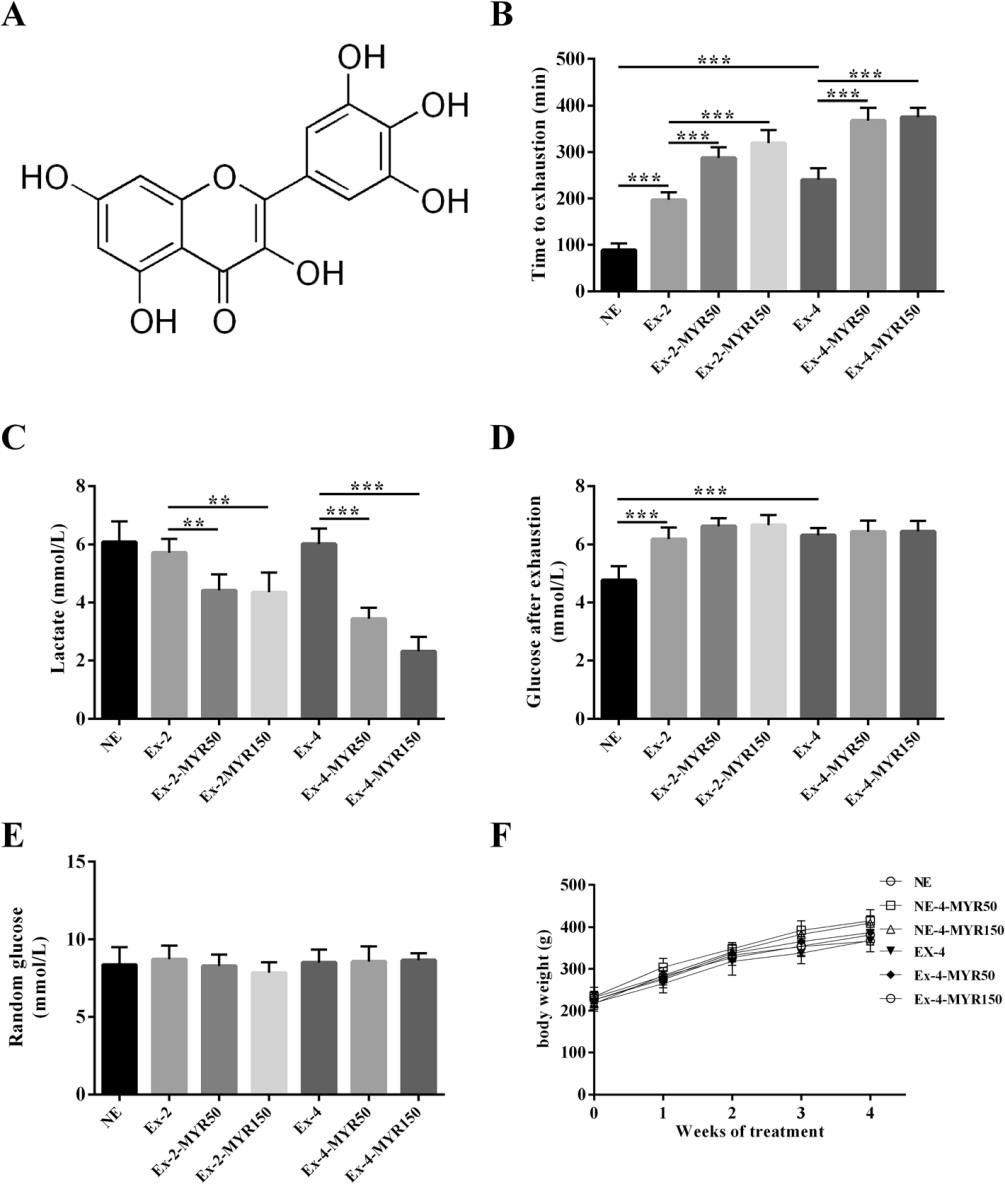
The effect of myricetin on endurance capacity in rats. **a** Chemical structures of myricetin. After the run-to-fatigue experiments, time to exhaustion (**b**) was recorded, while blood lactate (**c**) and glucose (**d**) levels were determined. Random glucose (**e**) and body weight (**f**) was monitored weekly. Data are presented as mean ± SD. **P<0.01, ***P<0.001

The chemical structure of myricetin, a natural flavonoid, was showed in Fig. 1a. Exercise indeed remarkably enhanced endurance capacity. As shown in Fig. 1b, TTE of both 2-week- and 4-week-exercise groups were significantly boosted, in a time-dependent way, compared with non-exercise group (*P* < 0.001). Intriguingly, both 2-week- and 4-week-treatment of myricetin exerted dramatic influence on the increase of TTE compared with exercise group (*P* < 0.001). Two weeks of treatments with low-dose (50 mg/kg) and high dose (150 mg/kg) myricetin were found to elevate TTE by 1.5-fold and 1.6-fold, respectively (*P* < 0.001), while four weeks of treatments with low-dose and high-dose myricetin increased TTE by 1.5-fold and 1.6-fold, respectively (*P* < 0.001).

**Fig. 2:**
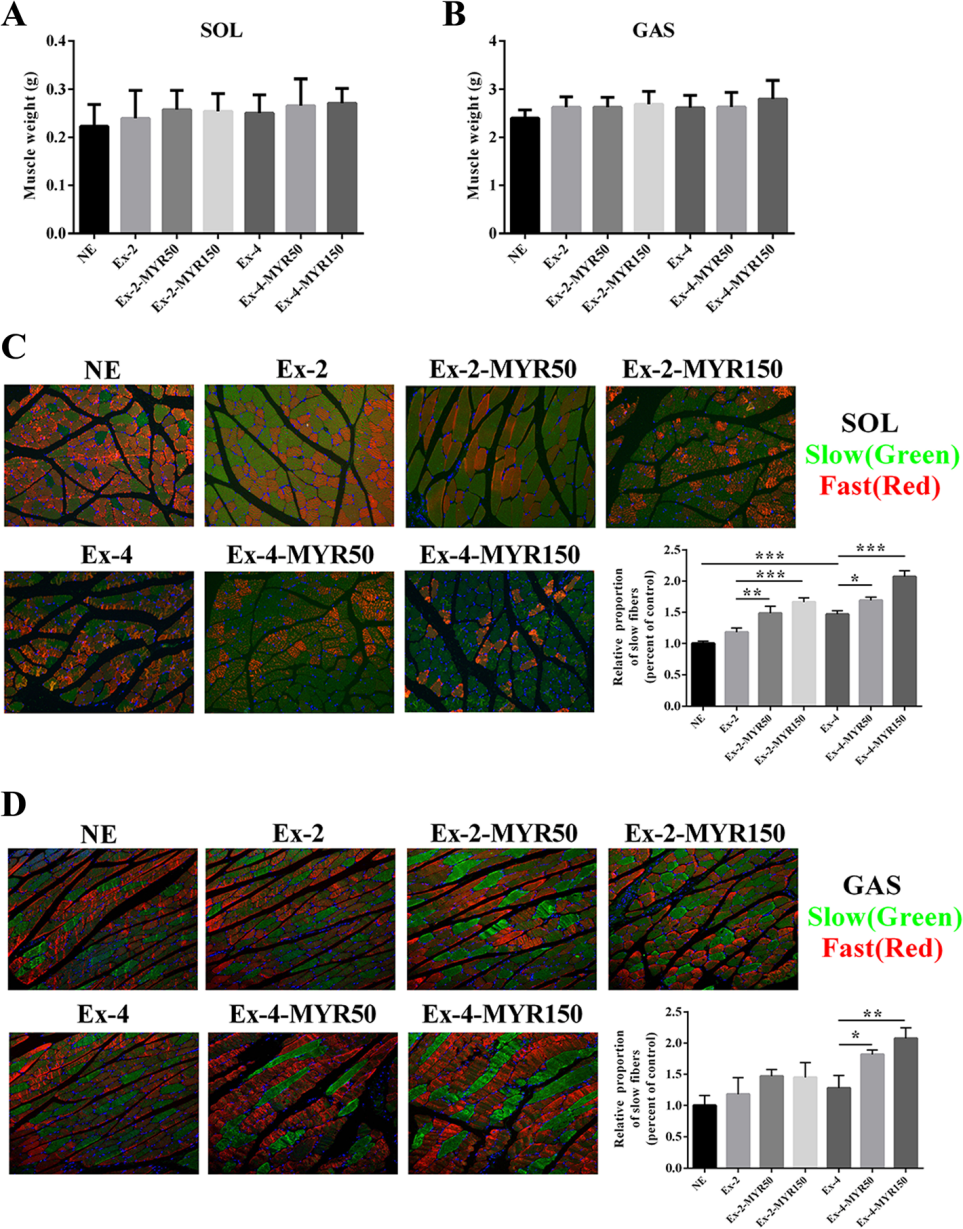
The effects of myricetin on the formation of slow muscle fibers in vivo. Soleus (**a**) and gastrocnemius (**b**) muscle mass was detected at the end of experiment. Soleus (**c**) and gastrocnemius (**d**) muscle phenotypes were shown by immunofluorescent staining. Data are presented as mean ± SD. *P<0.05, **P<0.01, ***P<0.001

Blood samples were collected after exhaustion. Exercise showed no significant effects on the level of lactate (*P* > 0.05 vs. non-exercise group, Fig. 1c), while both low-dose (50 mg/kg) and high-dose (150 mg/kg) of myricetin treatments remarkably reduced the levels of lactate (*P* < 0.01, Ex-2-MYR50 vs. Ex-2, Ex-2-MYR150 vs. Ex-2; *P* < 0.001, Ex-4-MYR50 vs. Ex-4, Ex-4-MYR150 vs. Ex-4, Fig. 1c). Interestingly, exercise significantly elevated plasma glucose after exhaustion compared with non-exercise group (*P* < 0.001, Fig. 1d), while myricetin treatments showed non-significant growth in levels of glucose after exhaustion compared with exercise groups (*P* > 0.05, Fig. 1d). Random plasma glucose among all groups was unchanged (*P* > 0.05, Fig. 1e), indicating that myricetin was nontoxic to glucose metabolism. These results, together with TTE data, suggested that myricetin enhanced endurance capacity in rats. The beneficial effects of myricetin on TTE did not result from decreased animal weight, since body weight did not differ among groups during both 4-week-experiment (Fig. 1f) and 2-week-experiment (data were not shown).

### Myricetin enhanced proportions of slow-twitch fibers in vivo

To investigate whether myricetin improved exercise performance of trained rats through enlarging the proportion of slow-twitch myofibers, we first evaluated the effect of myricetin on skeletal muscle mass and there were no differences between these groups (*P* > 0.05, Fig. 2a and b). Then we analyzed skeletal muscle fiber composition of soleus and gastrocnemius muscles. The results of immunofluorescence showed that myricetin dose-dependently increased the proportion of slow-twitch myofibers in both soleus (*P* < 0.01, Ex-2-MYR50 vs. Ex-2; *P* < 0.001, Ex-2-MYR150 vs. Ex-2; *P* < 0.05, Ex-4-MYR50 vs. Ex-4; *P* < 0.001, Ex-4-MYR150 vs. Ex-4, Fig. 2c) and gastrocnemius (*P* < 0.05, Ex-4-MYR50 vs. Ex-4; *P* < 0.01, Ex-4-MYR150 vs. Ex-4, Fig. 2d) muscles.

**Fig. 3:**
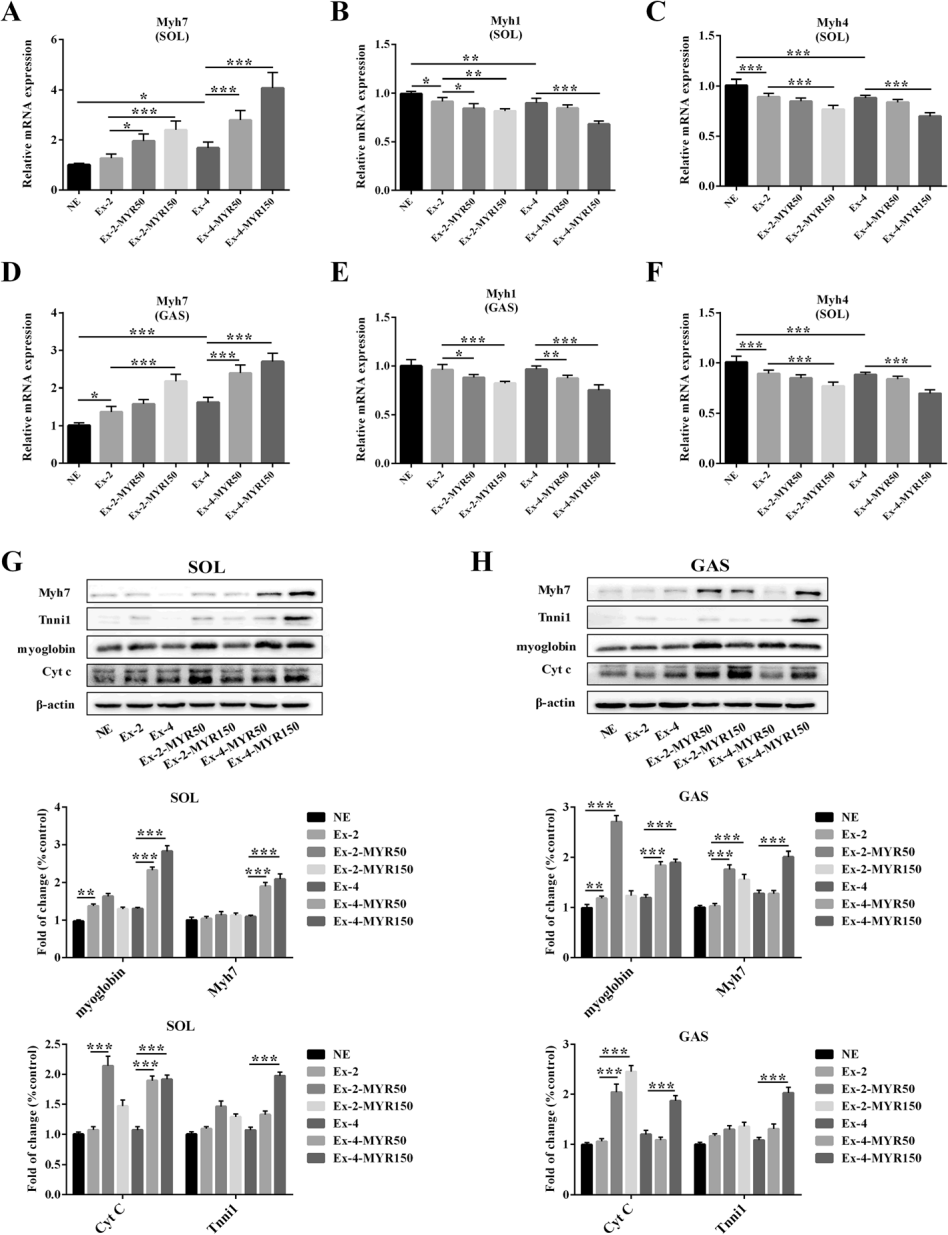
The effects of myricetin on mRNA and protein expressions of slow twitch-specific in vivo. The mRNA levels of slow-twitch myosin *Myh7* (**a**) and fast-twitch myosin *Myh1* (**b**), *Myh4* (**c**) in soleus and gastrocnemius (**d-f**) were determined by Real-time PCR. Protein levels of slow-twitch myosin Myh7 and slow-twitch fiber biomarkers in SOL (**g**) and GAS (**h**) were measured by immunoblotting. Data are presented as mean ± SD. *P<0.05, **P<0.01, ***P<0.001

Further more, mRNA expression of *Myh7* gene, predominantly expressed in slow-twitch myofibers, was increased in soleus and gastrocnemius muscles of myricetin-treated rats (Fig. 3a and d), concomitant with a decline in mRNA expression of *Myh1*, and *Myh4* genes, predominantly expressed in fast-twitch myofibers (Fig. 3b, c and e, f). The results of immunoblotting further confirmed that myricetin significantly increased protein expression of *Myh7*. Moreover, protein expressions of myoglobin, tnni1, and Cyt C, which are molecular markers of slow-twitch myofibers, were also elevated in myricetin-treated rats’ soleus and gastrocnemius muscles (Fig. 3g and h). These results suggested that myricetin increases the proportion of slow-twitch myofibers in trained rats.

### Myricetin promotes fast-to-slow switch in vitro

**Fig. 4:**
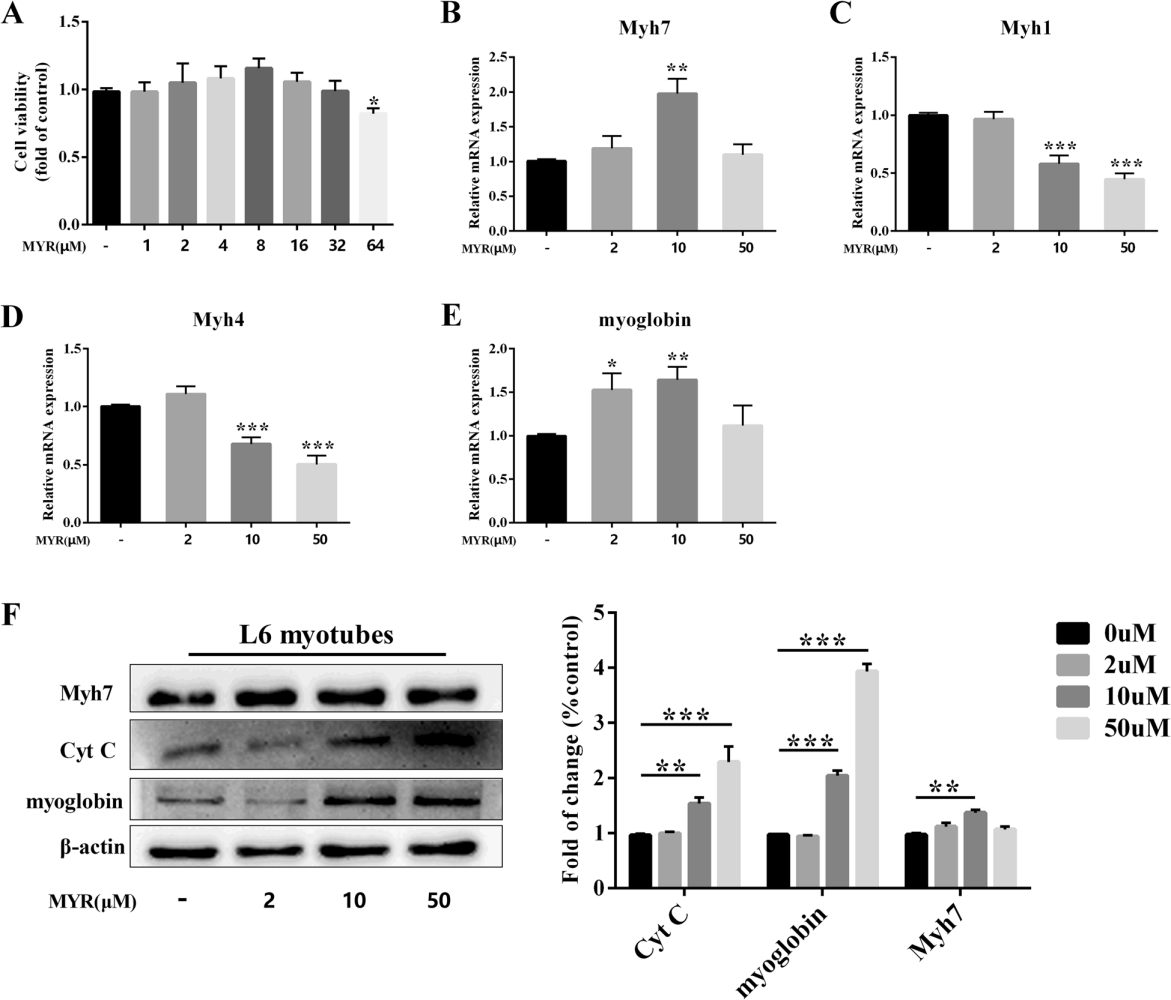
The effects of myricetin on muscle fiber-type composition in L6 myotubes. Cell viability (**a**) was measured using CCK-8 assay. The mRNA expressions of slow-twitch myosin *Myh7* (**b**), fast-twitch myosin *Myh1* (**c**), *Myh4* (**d**), and slow-twitch fiber biomarker myoglobin (**e**) were determined by real-time PCR. Protein levels of slow-twitch myosin *Myh7* and slow-twitch fiber biomarkers (**f**) in L6 myotubes were measured by immunoblotting. Data are presented as mean ± SD. *P<0.05, **P<0.01, ***P<0.001 compared with control

To further verify the role of myricetin in promoting the formation of slow-twitch myofibers, we next detected the effects of myricetin on fast-to-slow switch in rat L6 myotubes. Myricetin did not significantly suppress cell viability at concentrations lower than 64 μM (Fig. 4a). Myricetin at 10 μM significantly enhanced mRNA and protein expression of *Myh7* in L6 myotubes (*P* < 0.01, Fig. 4b and f), while high-dose (50 μM) showed no significant effect on promoting *Myh7* expression. Myricetin reduced mRNA expressions of fast-twitch myofiber genes, *Myh1* and *Myh4* (*P* < 0.001, Fig. 4c and d). Low- and middle-dose of myricetin treatments (2 μM and 10 μM, respectively) evidently increased mRNA expression of myoglobin, a molecular marker of slow-twitch myofiber (*P* < 0.05 and *P* < 0.01, respectively, Fig. 4e), although high-dose (50 μM) of myricetin showed no significant effect on elevating myoglobin expression. Protein levels of slow-twitch myofiber markers, myoglobin and Cyt C, were elevated by middle- and high-dose treatments of myricetin (10 μM and 50 μM, respectively), while low-dose treatment of myricetin (2 μM) showed no significant effect on protein levels of myoglobin and Cyt C (*P* > 0.05, Fig. 4f). Together with in vivo data, these results suggested that the effects of myricetin on fast-to-slow switch might contribute to enhancement of endurance capacity.

### Myricetin regulated miR-499/Sox6 in vivo and in vitro

**Fig. 5:**
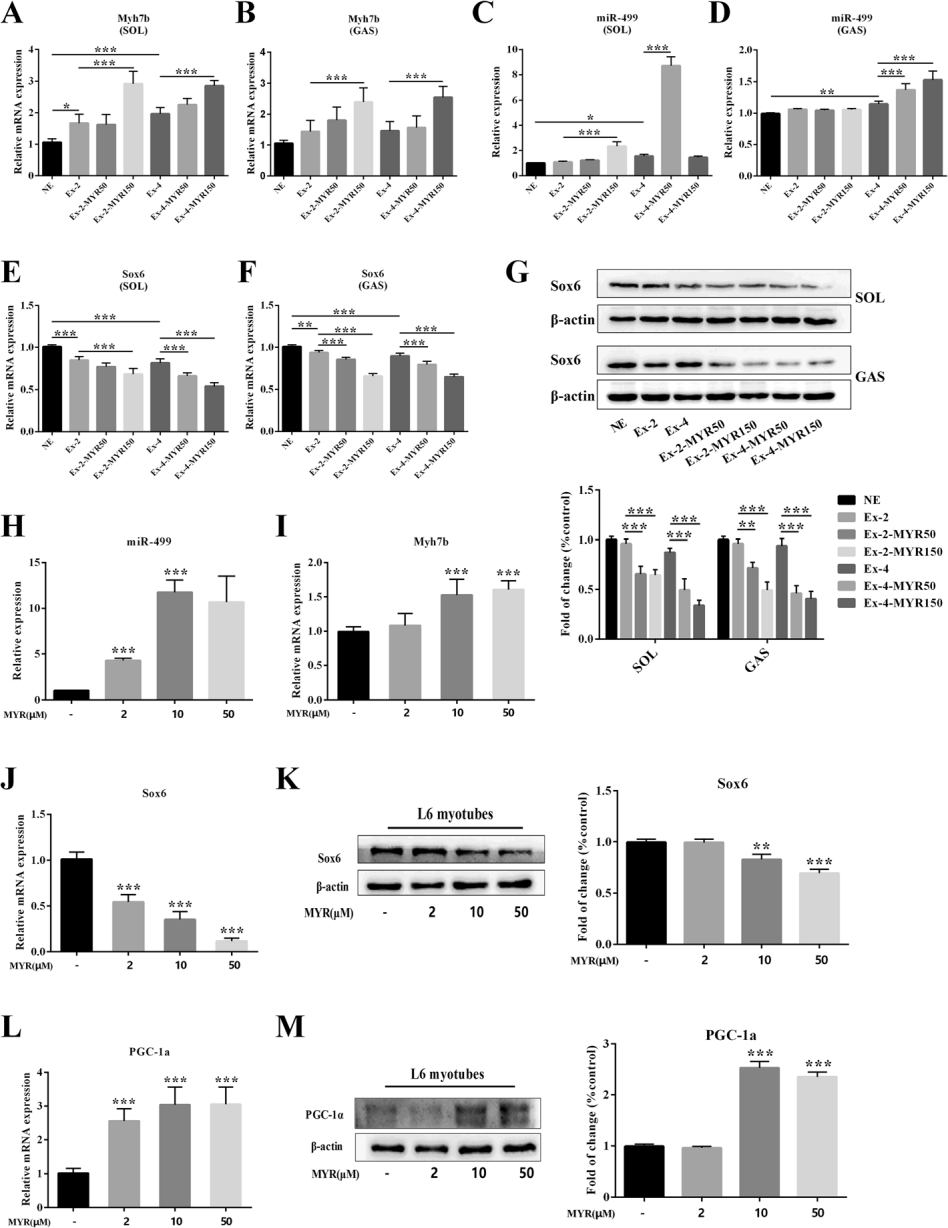
The effects of myricetin on regulating miR-499/Sox6 axis in vivo and in vitro. The mRNA expressions of *Myh7b* (**a**: soleus; **b**: gastrocnemius), miR-499 (**c**: soleus; **d**: gastrocnemius) and Sox6 (**e**: soleus; **f**: gastrocnemius) in rats were determined by Real-time PCR. Protein levels of Sox6 (**g**) in soleus and gastrocnemius muscles were measured by immunoblotting. The mRNA expressions of miR-499 (**h**), *Myh7b* (**i**), Sox6 (**j**), and PGC-1α (**l**) in L6 myotubes were determined by Real-time PCR. Protein levels of Sox6 (**k**) and PGC-1α (**m**) in L6 myotubes were measured by immunoblotting. Data are presented as mean ± SD. *P<0.05, **P<0.01, ***P<0.001

miR-499, encoded by *Myh7b* gene, plays a crucial role in reprogramming of fast-to-slow fiber type switch. Hence, we further explored whether myricetin promoted a fast-to-slow switch via regulating the expression of miR-499. High-dose (150 mg/kg) treatments of myricetin for short term (2 weeks) and long term (4 weeks) significantly up-regulated the expression of Myh7b both in soleus and gastrocnemius muscles (*P* < 0.001, Ex-2-MYR150 vs. Ex-2, Ex-4-MYR150 vs. Ex-4, Fig. 5a and b). Four-week treatment of myricetin elevated expression of miR-499 in gastrocnemius muscle (*P* < 0.001, Ex-4-MYR50 vs. Ex-4, Ex-4-MYR150 vs. Ex-4, Fig. 5d). In soleus muscle, high-dose (150 mg/kg) treatment of myricetin for 2 weeks evidently increased the expression of miR-499 (*P* < 0.001, Ex-2-MYR150 vs. Ex-2, Fig. 5c). However, as for long-term treatment, low-dose (50 mg/kg) myricetin dramatically boosted miR-499 expression (*P* < 0.001, Ex-4-MYR50 vs. Ex-4, Fig. 5c). Notably, we found that the mRNA and protein expression of Sox6, a transcriptional repressor of slow-twitch contractile protein genes, was markedly decreased both in soleus and gastrocnemius muscles after myricetin treatment (Fig. 5e-g).

To confirm the findings in vivo, we next examined the effects of myricetin in L6 myotubes. Myricetin dose-dependently elevated the mRNA expression of miR-499 and *Myh7b* (Fig. 5h and i). Meanwhile, mRNA and protein expression of Sox6 were markedly decreased (Fig. 5j and k). Additionally, myricetin markedly up-regulated the expression of both mRNA and protein of PGC-1α (Fig. 5l and m). Taken together, these data suggest that the effects of myricetin on regulating miR-499/Sox6 signaling pathway may contribute, at least in part, to the reprogramming of fast-to-slow fiber type switch.

### miR-499 is required for the myricetin effects on myofiber type conversion

**Fig. 6:**
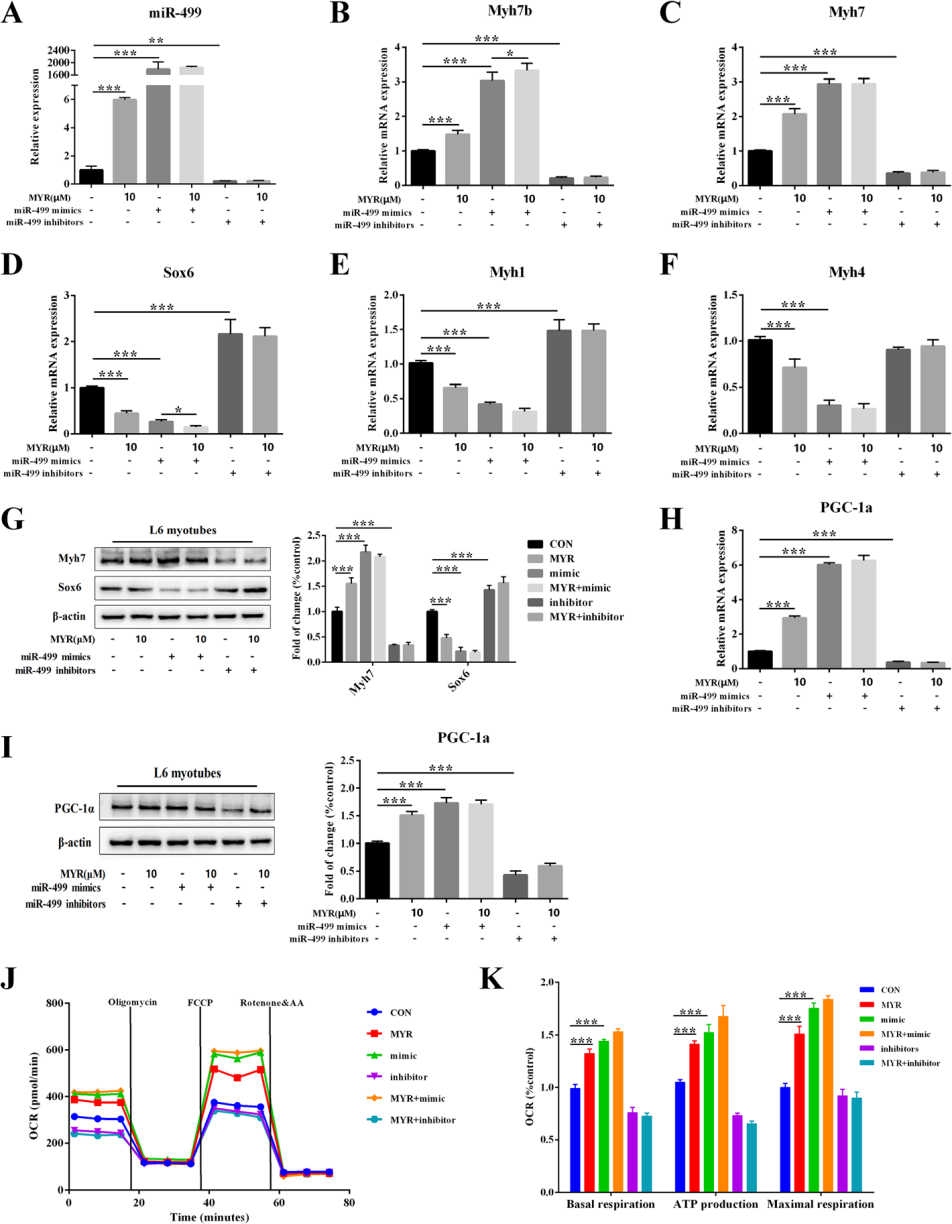
miR-499/Sox6 axis is involved in inducing fast-to-slow fiber type transition by myricetin. After transfected with inhibitor or mimic of miR-499, the mRNA expressions of miR-499 (**a**), *Myh7b* (**b**), *Myh7* (**c**), Sox6 (**d**), *Myh1* (**e**), and *Myh4* (**f**) in L6 myotubes were determined. Protein levels (**g**) of *Myh7* and Sox6 were measured by immunoblotting. The mRNA (**h**) and protein (**i**) levels of PGC-1α were monitored in the transfected L6 myotubes. Oxygen consumption rates (OCR) of myotubes (**j**) were determined by Seahorse XFp metabolic flux analysis. Basal respiration, ATP production and maximal respiration (**k**) were analyzed. Data are presented as mean ± SD. *P<0.05, **P<0.01, ***P<0.001

To verify the role of miR-499 in the effects of myricetin to promote myofiber type conversion, we transfected miR-499 inhibitor and mimic into L6 myotubes. The transfection of miR-499 inhibitor declined mRNA expression of miR-499 by 78.4% (*P* < 0.01, Fig. 6a). Overexpression of miR-499 resulted in markedly increases of mRNA expression of *Myh7b* and *Myh7*, and protein expression of *Myh7* in L6 myotubes (*P* < 0.001, Fig. 6b, c and g). Significant decreases of mRNA expression of Sox6, *Myh1*, and *Myh4* were spotted in L6 myotubes (*P* < 0.001, Fig. 6d, e and f). Intriguingly, compared with miR-499 mimic alone, myricetin together with miR-499 mimic acted robustly curtailed mRNA expression of Sox6 (*P* < 0.05, Fig. 6d). Furthermore, we investigated PGC-1α and mitochondrial respiration capacity in myotubes, and found that myricetin significantly amplified basal mitochondrial respiration oxygen consumption rate (OCR), ATP production, maximal respiration, and the expression of PGC-1α (Fig. 6h-k). Notably, inhibition of miR-499 overturned the effects of myricetin in L6 myotubes, indicating that miR-499 played a crucial role in the effects of myricetin on promoting the reprogramming of fast-to-slow fiber type switch.

## Discussion

In the present study, we found that natural flavonoid myricetin notably augmented endurance capacity of SD rats and increased the TTE treadmill running. Meanwhile, myricetin enhanced proportions of slow-twitch fibers in rats’ muscles. Interestingly, myricetin stimulated the expression of miR-499 both in vivo and in vitro, while inhibiting miR-499 could block the effects of myricetin on myotubes. Our results uncovered a novel mechanism that myricetin improved endurance capacity through promoting fast-to-slow myofiber transition via miR-499 pathway.

Skeletal muscle force can be distinctly modulated by exercise, as clearly shown in athletes. These adaptive changes, known as muscle plasticity, can be achieved by modifying the structural and functional properties of individual fibers or the proportion of distinct fiber populations in a muscle or a muscle group [5]. The present study showed that exercise indeed remarkably improved TTE treadmill running in SD rats. Interestingly, our in vivo data also showed that myricetin treatment potently amplified endurance capacity by remarkably increasing TTE. Previous studies have demonstrated that natural compounds, such as quercetin and resveratrol, could enhance endurance capacity [22, 23]. Meanwhile, we found that myricetin remarkably reduced the levels of lactate after exhaustion treadmill running. It is well accepted that muscle metabolic changes, such as lactate accumulation, are involved in the development of muscle fatigue [24]. Hence, myricetin enhanced anti-fatigue ability of rats, which might contribute, at least in part, to the increase of exercise endurance.

Several lines of evidence have demonstrated that long-term endurance training induced an increased content of capillaries and mitochondria in skeletal muscle, promoted muscle fiber phenotype fast-to-slow (type II to typeI) transition, and thereby improved endurance capacity [25]. Recently, Mizunoya et al. reported that apple polyphenols improved endurance by inducing fast-to-slow shift of muscle fiber type composition [26, 27]. We expect that myricetin, also a flavonoid, might promote muscle fiber type transition, which led to endurance enhancement. And, in the present study we detected that myricetin dose-dependently increased the proportion of slow-twitch myofibers in both soleus and gastrocnemius muscles. Moreover, our in vitro data showed that myricetin up-regulated the expression of *Myh7* gene, predominantly expressed in slow-twitch myofibers, and down-regulated the expression of *Myh1*, and *Myh4* genes, predominantly expressed in fast-twitch myofibers, which further demonstrate this point. Therefore, we propose that myricetin improves exercise endurance through promoting reprogramming of fast-to-slow fiber type switch.

MyomiRs regulate myosin switching and myofiber identity. Eva van Rooij et al. demonstrated that the miR-499 transgenic mice ran more than 50% longer than wild-type littermates, indicating improved endurance due to the fast to slow myofibers reprogramming [1]. Notably, although miR-499 was found encoded by mouse *Myh7b* gene, it is relevant in human. Animal and human researches revealed that miR-499 expression were higher in muscles of trained “active” individuals compared with those of sedentary individuals. Moreover, a strong positive correlation was observed between miR-499’s expression and type I myofiber percentage, ATPmax and VO_2_max [28]. In accordance with these findings, our study found that myricetin remarkably augmented the expression of miR-499 and *Myh7b* both in vivo and in vitro, which further expounded how myricetin regulate the reprogramming of fast-to-slow fiber type switch.

The function of miR-499 on regulating gene expression is exerted by down-regulation of transcriptional repressors, such as Sox6 that suppresses slow-twitch contractile protein gene expressions. Researches found that miR-499 directly bound Sox6 and suppressed its expression, and eventually promoted type I fiber formation [29, 30]. In the present study, we investigated the effects of myricetin on regulating miR-499/Sox6 axis and found that myricetin significantly reduced Sox6 expression along with increase of miR-499 expression. Intriguingly, inhibition of miR-499 overturned the effects of myricetin on the suppression of Sox6, indicating that myricetin exerts its beneficial effects on muscle fiber type switch through the miR-499/Sox6 pathway. As to the precise mechanism for the regulation of miR-499 by myricetin, there are some researches might shed some light on the matter. Taken together, there are two possibilities of the underlying mechanism. First, myricetin might directly bind to miR-499 to exert its effects. Eshan Khan and colleagues found that myricetin was a potent and selective binder of 5’CAG/3’GAC motif containing RNA, and interacted with this RNA via base stacking at AA mismatch [31]. The stem-loop structure of pre-miR-499 also has a 5’CAG/3’GAC motif. Hence, myricetin might directly bind at this site of pre-miR-499. Second, myricetin might regulate the up-stream nuclear receptors, such as peroxisome proliferator-activated receptors (PPARs), to affect the expression of miR-499. The ligand-binding activation of PPARβ/δ induced fast-to-slow muscle fiber type switch and increased the muscle oxidative capacity [32]. Many natural phytochemicals, such as green tee and bitter melon extracts, are found to activate PPARβ/δ to improve insulin sensitivity [33, 34]. Myricetin, as a natural flavonoid, might activate PPARβ/δ to regulate slow-twitch myofiber related gene expressions. However, efforts are needed to elucidate the underlying mechanism.

The main character of slow-twitch fibers is their high contents of mitochondria and oxidative enzymes. Intriguingly, we found that myricetin elevated oxygen consumption rates in L6 myotubes, indicating an increase of ATP production and max respiration. Inhibition of miR-499 attenuated the beneficial effects of myricetin on oxidative metabolism. Moreover, the nuclear receptor coactivator PGC-1α (PPARγ coactivator-1α) was up regulated after myricetin treatment and down regulated when miR-499 was curbed. As we know, PGC-1α is a downstream transcriptional regulator of AMPK (AMP-activated protein kinase), key sensor of cellular metabolic and energy homeostasis, which plays a crucial role in skeletal muscle oxidative metabolism and fiber-type specification [35–37]. Recently, Liu Jing et al. demonstrated that miR-499 repressed expression of Fnip1 (folliculin-interacting protein-1) by directly binding, and therefore activated downstream AMPK/PGC-1α signaling pathway [38]. Taken together, our data showed that myricetin improved muscle oxidative metabolism by elevating expression of miR-499 and subsequently activated PGC-1α signaling pathway.

In summary, our current study has further elucidated the mechanism underlying the effect of myricetin. To the best of our knowledge, this study provides the first evidence that myricetin improves endurance capacity by inducing muscle fiber type transition, and microRNA miR-499 plays an important role in the effect of myricetin. Although more work is needed to elucidate the intermolecular interactions, our findings suggest that myricetin is a potential candidate to improve endurance capacity.

## Conclusions

Skeletal muscle is essential both to exert physical performance and maintain energy homeostasis. Certain stimuli, like endurance exercise, can induce fast-to-slow muscle fiber type switch, and subsequently improve aerobic capacity and metabolic status. In the current study, we showed that myricetin improved endurance capacity by regulating fast-to-slow muscle fiber type conversion. In addition, the in vivo and in vitro experiments showed that this function of myricetin was associated with miR-499. Our findings have important implications for improving the health of sedentary people and people with muscle diseases.

## Abbreviations

AMPK: Adenosine 5′-monophosphate activated protein kinase
ATP: Adenosine triphosphate
BSA: Bovine serum albumin
CCK-8: Cell Counting Kit-8
DAPI: 4′,6-diamidino-2-phenylindole
DMEM: Dulbecco’s Modified Eagle’s medium
DMSO: Dimethyl sulfoxide
DNA: Deoxyribonucleic acid
EDTA: Ethylenediaminetetraacetic acid
FCCP: Carbonyl cyanide-p-trifluoromethoxyphenylhydrazone
GAPDH: Glyceraldehyde-3-phosphate dehydrogenase
GAS: Gastrocnemius
GLUT4: Glucose transporter type 4
HRP: Horseradish Peroxidase
MHC: myosin heavy chain
Myh: Myosin heavy chain
Myh1: Myosin heavy chain1
Myh4: Myosin heavy chain4
Myh7: Myosin heavy chain7
Myh7b: Myosin heavy chain7b
NRF-1: Nuclear respiratory factor 1
OCR: Oxygen consumption rate
PBS: Phosphate buffered saline
PCR: Polymerase chain reaction
PGC-1α: Peroxlsome proliferator-activated receptor-γ coactlvator-1α
PPARβ: Peroxisome Proliferator-Activated Receptor β
PVDF: Polyvinylidene fluoride
qRT-PCR: Quantitative real time polymerase chain reaction
RNA: Ribonucleic acid
SD: Sprague Dawley
SDS-PAGE: Sodium dodecyl sulfate-polyacrylamide gel electrophoresis
SOL: Soleus
Sox6: Sex-determining region Y-box 6
TNNI1: Troponin I type 1
TTE: Time to exhaustion
