# Correction to: Myricetin improves endurance capacity by inducing muscle fiber type conversion via miR-499

**DOI:** 10.1186/s12986-021-00572-1

**Published:** 2021-06-03

**Authors:** Luting Wu, Li Ran, Hedong Lang, Min Zhou, Li Yu, Long Yi, Jundong Zhu, Lei Liu, Mantian Mi

**Affiliations:** Research Center for Nutrition and Food Safety, Chongqing Key Laboratory of Nutrition and Food Safety, Institute of Military Preventive Medicine, Third Military Medical University, Chongqing, China

## Correction to: Wu et al. Nutr Metab (Lond) (2019) 16:27 https://doi.org/10.1186/s12986-019-0353-8

Following publication of the original article [1], the authors identified errors in Figs. 2 and 3. The correct figures are given below.

**Fig. 2:**
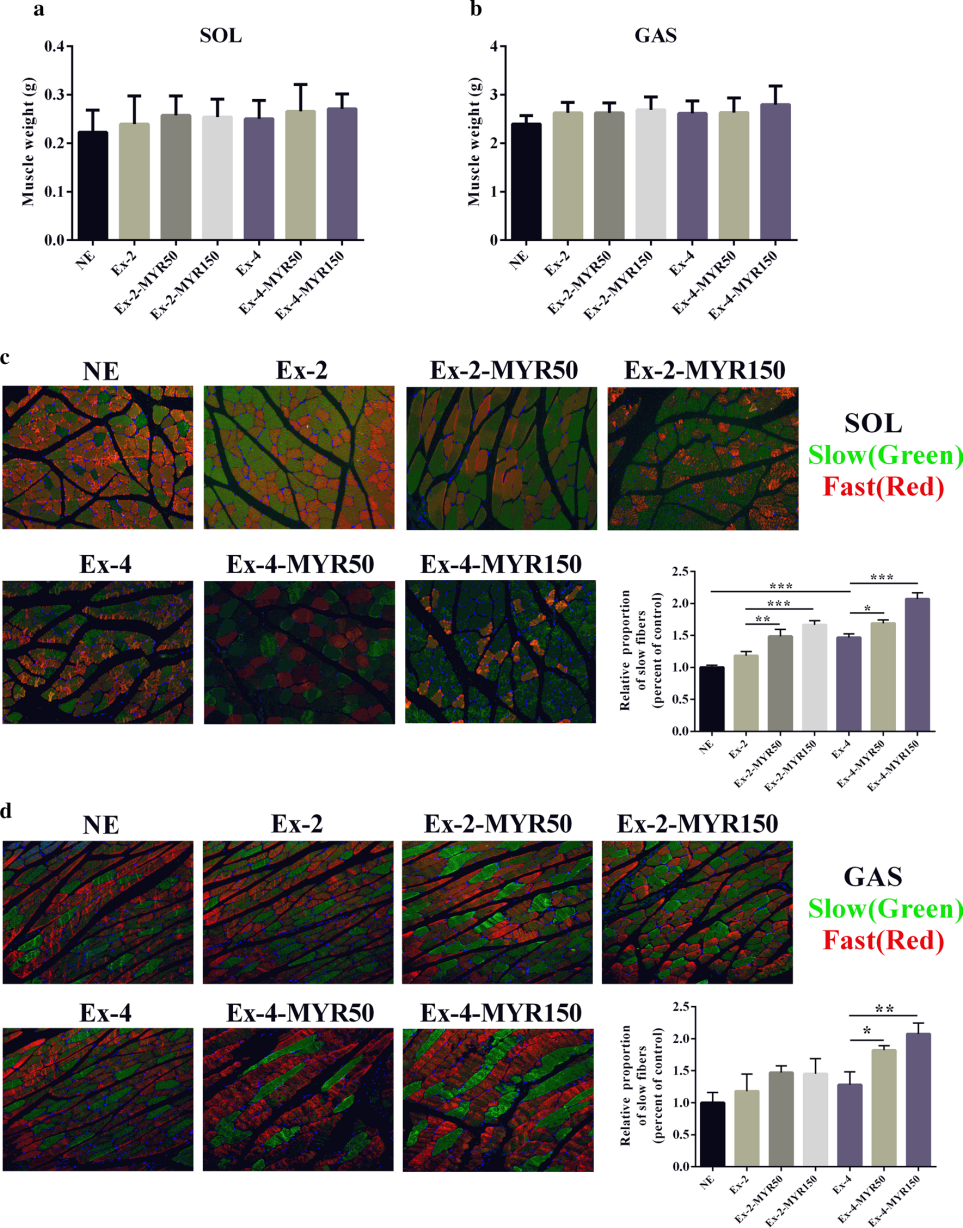
The effects of myricetin on the formation of slow muscle fibers in vivo. Soleus (**a**) and gastrocnemius (**b**) muscle mass was detected at the end of experiment. Soleus (**c**) and gastrocnemius (**d**) muscle phenotypes were shown by immunofluorescent staining. Data are presented as mean ± SD. *P < 0.05, **P < 0.01, ***P < 0.001

**Fig. 3:**
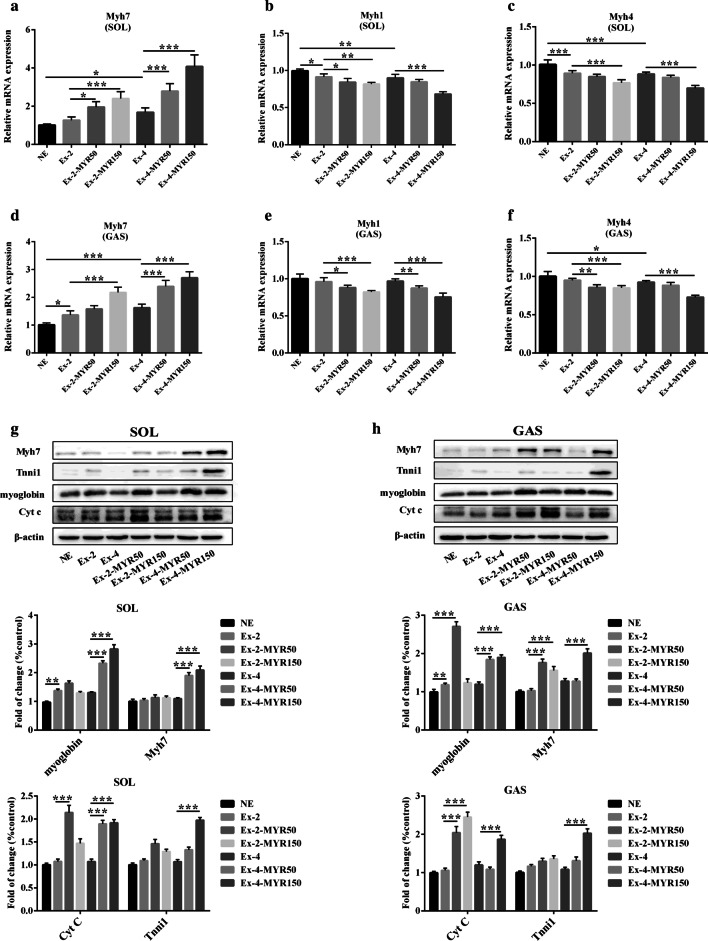
The effects of myricetin on mRNA and protein expressions of slow twitch-specific in vivo. The mRNA levels of slow-twitch myosin *Myh7* (**a**) and fast-twitch myosin *Myh1* (**b**), *Myh4* (**c**) in soleus and gastrocnemius (**d**–**f**) were determined by Real-time PCR. Protein levels of slow-twitch myosin Myh7 and slow-twitch fiber biomarkers in SOL (**g**) and GAS (**h**) were measured by immunoblotting. Data are presented as mean ± SD. *P < 0.05, **P < 0.01, ***P < 0.001

The author group has been updated above and the original article [1] has been corrected.
